# Dexamethasone vs. Dexmedetomidine as Adjuvants to Erector Spinae Plane Block in Total Knee Arthroplasty: A Randomized Double-Blind Controlled Trial

**DOI:** 10.3390/jcm15124513

**Published:** 2026-06-11

**Authors:** Ewa Grelowska, Tomasz Reysner, Jowita Rosada-Kurasińska, Justyna Marszałek-Buko, Paweł Pietraszek, Anna Perek, Aleksandra Łakomy, Katarzyna Wieczorowska-Tobis, Przemysław Daroszewski, Malgorzata Reysner

**Affiliations:** 1 Department of Anesthesiology and Intensive Care, Poznan University of Medical Sciences, ul. Długa 1/2, 61-848 Poznan, Poland; 2 Pathophysiology of Pain Unit, Department of Anesthesiology and Intensive Care, Poznan University of Medical Sciences, ul. Przybyszewskiego 49, 60-355 Poznan, Poland; treysner@ump.edu.pl; 3 Department of Pediatric Anesthesiology and Intensive Care, Poznan University of Medical Sciences, ul. Szpitalna 27/33, 60-572 Poznan, Poland; 4 Department of Clinical Anesthesiology and Pain Management, Poznan University of Medical Sciences, ul. 28 Czerwca 1956 135/147, 61-701 Poznan, Poland; 5 Department of Palliative Medicine, Poznan University of Medical Sciences, ul. Os. Rusa 55, 61-245 Poznan, Poland; 6 Department of Pathophysiology of Locomotor Organs, Wiktor Dega Orthopedic Institute, Poznan University of Medical Sciences, ul. 28 Czerwca 1956 r. 135/147, 61-545 Poznan, Poland

**Keywords:** erector spinae plane block, dexamethasone, dexmedetomidine, total knee arthroplasty, postoperative analgesia, opioid consumption, regional anesthesia, randomized controlled trial

## Abstract

**Background**: Total knee arthroplasty is associated with significant postoperative pain. The erector spinae plane block (ESPB) is increasingly used as part of multimodal analgesia, but its duration may be limited. Adjuvants such as dexamethasone and dexmedetomidine may enhance analgesic efficacy; however, direct comparisons between these agents in ESPB remain limited. **Methods**: In this prospective, randomized, double-blind trial, 90 patients undergoing total knee arthroplasty were allocated to receive ESPB with ropivacaine alone (control), with ropivacaine plus dexamethasone (DEX), or with ropivacaine plus dexmedetomidine (DEM) (*n* = 30 per group). The primary outcome was time-to-first opioid requirement within 48 h. Secondary outcomes included total opioid consumption, postoperative pain intensity (NRS), and adverse events. Time-to-event analysis was performed using Kaplan–Meier and Cox regression. **Results**: Time-to-first opioid requirement was significantly prolonged in both DEX and DEM groups compared with control (log-rank *p* < 0.0001). Median time was 7.85 h (95% CI 7.40–8.50) in control, 13.70 h (12.80–14.50) in DEX, and 12.40 h (11.20–14.30) in DEM. Both DEX (HR 0.036, 95% CI 0.017–0.079) and DEM (HR 0.058, 95% CI 0.028–0.121) significantly reduced the hazard of opioid requirement (*p* < 0.0001). Total opioid consumption was significantly lower in the DEX group compared with both control and DEM (*p* < 0.0001), while no difference was observed between DEM and control. Pain scores were lower in the DEX group in the early postoperative period. No statistically significant differences in adverse events were observed between groups. **Conclusions**: Both dexamethasone and dexmedetomidine prolong ESPB analgesia in total knee arthroplasty. However, dexamethasone provides superior analgesic efficacy without increasing adverse events.

## 1. Introduction

Total knee arthroplasty is associated with significant postoperative pain, which may negatively affect early mobilization, rehabilitation, and overall recovery [1]. Effective postoperative analgesia is therefore a key component of perioperative care, and multimodal analgesic strategies, including regional anesthesia techniques, are widely recommended to reduce opioid consumption and improve outcomes [2].

Despite advances in perioperative pain management, optimal analgesia following total knee arthroplasty remains challenging [3]. Postoperative pain after TKA is often severe and may impair early mobilization, delay rehabilitation, prolong hospital stay, and negatively affect patient satisfaction and functional recovery [4]. Furthermore, inadequate pain control may contribute to persistent postoperative pain and increased opioid consumption, which remains an important clinical concern in orthopedic surgery [5].

Regional anesthesia techniques have therefore become an essential component of enhanced recovery after surgery (ERAS) pathways in total knee arthroplasty. Several approaches have been investigated, including femoral nerve block, adductor canal block, iPACK block, periarticular infiltration, and more recently, erector spinae plane block. Compared with traditional peripheral nerve blocks, ESPB may offer technical simplicity, a lower risk of motor weakness, and a favorable safety profile due to its distance from major neurovascular structures.

Although adductor canal block, femoral triangle block, iPACK block, and periarticular infiltration remain commonly used motor-sparing techniques for total knee arthroplasty, lumbar ESPB has emerged as a potentially simpler fascial-plane technique with a favorable safety profile and a possible multi-dermatomal spread. Nevertheless, the exact mechanism and extent of local anesthetic spread following lumbar ESPB remain incompletely understood.

Although ESPB has shown promising analgesic efficacy in lower-limb surgery, variability in block duration and postoperative analgesic requirements remains a limitation. Consequently, increasing attention has been directed toward the use of perineural adjuvants to prolong block duration and improve postoperative analgesia. Among the most commonly used adjuvants are dexamethasone and dexmedetomidine, both of which have demonstrated the ability to enhance peripheral nerve block characteristics in multiple clinical settings.

The erector spinae plane block (ESPB) has gained increasing popularity as a relatively simple and safe regional anesthesia technique. Although initially described for thoracic analgesia, its use has expanded to various surgical procedures, including lower-limb surgery [6]. However, the analgesic efficacy of ESPB in knee arthroplasty remains variable, and its clinical usefulness may be limited by the duration of action of local anesthetics [7].

To prolong the duration and improve the quality of regional blocks, various adjuvants have been investigated [8]. Among them, dexamethasone and dexmedetomidine are widely used and have been shown to enhance analgesia through different mechanisms. Dexamethasone is thought to prolong analgesia through anti-inflammatory effects, inhibition of nociceptive transmission, and vasoconstriction, whereas dexmedetomidine acts primarily via α2-adrenergic receptor-mediated modulation of pain pathways [9]. Despite growing evidence supporting their use, direct comparisons between these two agents as perineural adjuvants, particularly in ESPB for knee arthroplasty, remain limited [10].

However, evidence directly comparing dexamethasone and dexmedetomidine as adjuvants to lumbar ESPB in total knee arthroplasty remains scarce. Moreover, previous studies have primarily focused on peripheral nerve blocks, such as the adductor canal block and the femoral nerve block, while data on fascial-plane blocks remain limited [11,12]. Therefore, further randomized controlled trials are needed to clarify which adjuvant provides superior analgesic efficacy and safety in this clinical setting.

We hypothesized that both dexamethasone and dexmedetomidine would prolong analgesia compared with ESPB alone, but that dexamethasone would provide superior analgesic efficacy, as reflected by a longer time-to-first opioid requirement, lower opioid consumption, and improved early postoperative pain control.

Therefore, the aim of this study was to compare the effects of perineural dexamethasone and dexmedetomidine on the duration and quality of analgesia following ESPB in patients undergoing total knee arthroplasty.

## 2. Methods

### 2.1. Study Design and Participants

This prospective, randomized, double-blind, controlled trial was conducted at Poznan University of Medical Sciences, Poland, following approval from the local Bioethics Committee (approval number: 107/24). The study was registered on ClinicalTrials.gov (NCT06470100). The study protocol was developed in accordance with CONSORT recommendations for randomized controlled trials. A total of 90 adult patients scheduled for elective primary total knee arthroplasty were enrolled between August 2024 and March 2026. Written informed consent was obtained from all participants before inclusion. Inclusion criteria included age between 18 and 100 years and American Society of Anesthesiologists (ASA) physical status I–III. Patients were excluded if they declined participation, had a history of opioid abuse, infection at the injection site, severe renal or hepatic failure, coagulopathy, allergy to study drugs, pre-existing neurological deficits in the lower limbs, or inability to assess pain.

### 2.2. Randomization and Blinding

Patients were randomly allocated in a 1:1:1 ratio to one of three study groups (*n* = 30 per group) using a computer-generated randomization sequence created with randomizer.org. A block randomization scheme with a fixed block size of six was used to ensure balanced group allocation throughout the study.

Allocation concealment was ensured using sequentially numbered, sealed, opaque envelopes prepared by an independent investigator not involved in patient recruitment, intervention delivery, or outcome assessment. Envelopes were opened only after patient enrolment, immediately prior to preparation of the study solution.

The study was conducted under quadruple-blind conditions. Patients, anesthesiologists responsible for intraoperative management, investigators assessing postoperative outcomes, and statisticians performing the analysis were all blinded to group allocation.

Study medications were prepared by an independent anesthesiologist not involved in patient care or data collection. All study solutions were prepared in identical syringes with the same total volume and appearance to ensure blinding. The syringes were labelled only with the study identification number, without indication of group allocation.

The anesthesiologist performing the erector spinae plane block was blinded to the contents of the syringe. Blinding was maintained until completion of data analysis.

### 2.3. Interventions

All patients received an ultrasound-guided erector spinae plane block (ESPB) following the induction of spinal anesthesia and prior to the start of surgery.

Patients were allocated to one of three groups:

In the control group, 20 mL of 0.2% ropivacaine mixed with 2 mL of 0.9% saline was administered. In the dexamethasone group, 20 mL of 0.2% ropivacaine with 4 mg of dexamethasone was administered. In the dexmedetomidine group, 20 mL of 0.2% ropivacaine with 50 µg of dexmedetomidine was administered. The ESPB was performed under sterile conditions using a high-frequency linear ultrasound transducer. The block was performed at the L1 vertebral level, with the local anesthetic solution injected into the fascial plane deep to the erector spinae muscle under real-time ultrasound guidance.

### 2.4. Ultrasound-Guided ESPB Technique

All blocks were performed by experienced anesthesiologists with extensive expertise in ultrasound-guided regional anesthesia. Patients were placed in the sitting position. A high-frequency linear ultrasound transducer (6–13 MHz) covered with a sterile sheath was positioned in a longitudinal parasagittal orientation approximately 3 cm lateral to the spinous process at the L1 vertebral level. The transverse process and overlying erector spinae muscle were identified. After skin disinfection and local infiltration with 1% lidocaine, a 22-gauge, 80 mm echogenic block needle (Stimuplex^®^ Ultra 360, B. Braun Melsungen AG, Melsungen, Germany) was advanced in-plane in a cranial-to-caudal direction until contact with the transverse process was achieved. Correct needle placement within the fascial plane deep to the erector spinae muscle was confirmed by hydrodissection with 1–2 mL of saline. Subsequently, the study solution was injected incrementally under continuous ultrasound visualization to ensure adequate linear spread beneath the erector spinae muscle fascia. All blocks were performed unilaterally on the operative side immediately after spinal anesthesia and before surgical incision.

### 2.5. Anesthesia and Perioperative Management

Premedication was not routinely administered. Upon arrival in the operating room, standard monitoring was applied, including electrocardiography, non-invasive blood pressure, and pulse oximetry.

Spinal anesthesia was performed under aseptic conditions at the L3–L4 interspace using a midline approach. A total of 3–4 mL of 0.5% ropivacaine was administered intrathecally.

Sedation during surgery was provided with intravenous midazolam (1–3 mg) or propofol infusion at the discretion of the attending anesthesiologist. Sedation was titrated to maintain patient comfort while preserving spontaneous ventilation and verbal responsiveness. No significant between-group differences in intraoperative sedation requirements or postoperative excessive sedation were clinically observed. No long-acting opioids were administered intraoperatively.

Following induction of spinal anesthesia, all patients received an ultrasound-guided erector spinae plane block as described above.

Postoperative pain management followed a standardized multimodal analgesia protocol. All patients received intravenous paracetamol 1 g every 6 h and intravenous ketoprofen 100 mg every 12 h, unless contraindicated.

Rescue analgesia consisted of intravenous morphine administered in 2–3 mg boluses when the Numerical Rating Scale (NRS) score was ≥4, with a lockout interval of 10 min as needed.

### 2.6. Outcome Measures

The primary outcome of the study was the time-to-first opioid requirement. This was defined as the time interval, expressed in hours, from the completion of surgery to the first administration of rescue opioid analgesia within the first 48 h postoperatively.

### 2.7. Secondary Outcomes

Secondary outcomes included total opioid consumption, postoperative pain intensity, neurological complications, and the incidence of adverse events.

Total opioid consumption was measured cumulatively over the first 48 h postoperatively and expressed as intravenous morphine equivalents (mg).

Postoperative pain intensity was assessed using the Numerical Rating Scale (NRS), where 0 represented no pain and 10 represented the worst imaginable pain. Pain scores were recorded at predefined time points: 4, 8, 12, 16, 20, 24, 36, and 48 h after surgery.

Neurological outcomes were evaluated using a standardized nerve injury score at 12, 24, and 48 h postoperatively. The scoring system was defined as follows: N0, no neurological deficit; N1, minor sensory disturbance (paresthesia); N2, complete sensory loss; N3, complete motor deficit with or without sensory disturbance; and N4, complex regional pain syndrome.

In addition, adverse events were systematically recorded throughout the postoperative period. These included hemodynamic complications (hypotension and bradycardia), postoperative nausea and vomiting (PONV), excessive sedation, and any other clinically significant events potentially related to the study interventions or analgesic protocol.

Postoperative assessments were performed by investigators blinded to group allocation. Pain intensity was evaluated both at rest and during passive knee flexion using the Numerical Rating Scale (NRS). Hemodynamic parameters, opioid consumption, and adverse events were recorded at predefined postoperative time points throughout the 48 h observation period.

### 2.8. Sample Size Calculation

Sample size estimation was based on the primary endpoint, namely the time from the end of surgery to the first administration of rescue opioid analgesia within 48 h postoperatively. Because the primary analysis treated this endpoint as a time-to-event variable, the log-rank test was used for the calculation.

Before the definitive trial, a pilot study was undertaken in 18 patients (6 per group). These patients were enrolled solely to assess feasibility and to provide preliminary estimates of the treatment effect and event rate, and were not included in the final analysis. The pilot study was conducted at the same institution, using the same study protocol, perioperative management, investigators, and eligibility criteria as the definitive trial. In the pilot phase, the median time-to-first rescue opioid administration was 8.0 h in the control group, 18.0 h in the dexamethasone group, and 22.0 h in the dexmedetomidine group.

To ensure a conservative approach, the calculation was based on the smaller between-group difference, namely that between the dexamethasone and control groups. Under the assumption of an exponential survival distribution, this yielded an estimated hazard ratio of 0.44. Using a two-sided alpha of 0.05 and a power of 80%, 46.6 events were required. Given an anticipated event rate of 90% during the 48 h follow-up period, the corresponding minimum sample size was 52 patients.

The final sample size was increased to 90 patients to maintain balanced allocation across the three study groups and to safeguard against the effects of censoring, protocol deviations, and possible incomplete outcome data. Accordingly, 30 patients were assigned to each group.

### 2.9. Statistical Analysis

All perioperative and postoperative data were recorded using standardized case report forms and subsequently entered into a secure electronic database. Data accuracy was independently verified by two investigators prior to statistical analysis.

Statistical analysis was performed using GraphPad Prism 11.0.0 (GraphPad Software LLC, Boston, MA, USA). Continuous variables were assessed for normality using the Shapiro–Wilk test and are presented as mean (standard deviation) or median (interquartile range), as appropriate. Categorical variables are presented as numbers (percentages).

Baseline characteristics were compared between groups using one-way analysis of variance (ANOVA) for normally distributed continuous variables, the Kruskal–Wallis test for non-normally distributed continuous variables, and the chi-square test or Fisher’s exact test for categorical variables, as appropriate.

The primary endpoint, defined as the time from the end of surgery to the first administration of rescue opioid analgesia within 48 h postoperatively, was analysed as a time-to-event variable. Time-to-event curves were constructed using the Kaplan–Meier method and compared between groups using the log-rank test. All patients required rescue opioid analgesia during the observation period; therefore, no censoring occurred in the final survival analysis. Hazard ratios with 95% confidence intervals were estimated using Cox proportional hazards regression, with the control group used as the reference category.

Total opioid consumption during the first 48 postoperative hours was compared between groups using one-way ANOVA or the Kruskal–Wallis test, depending on data distribution. When the overall comparison was statistically significant, post hoc pairwise comparisons were performed with Bonferroni correction.

Postoperative pain intensity, assessed using the Numerical Rating Scale (NRS) at predefined time points, was analyzed using a two-way repeated-measures ANOVA with group as the between-subjects factor and time as the within-subjects factor. The Geisser–Greenhouse correction was applied when the sphericity assumption was not met. When significant group, time, or interaction effects were identified, post hoc multiple comparisons with correction for multiple testing were performed. Neurological outcomes and adverse events were analysed as categorical variables and compared between groups using the chi-square test or Fisher’s exact test, as appropriate.

All tests were two-sided, and a *p*-value < 0.05 was considered statistically significant.

## 3. Results

### 3.1. Patient Flow and Baseline Characteristics

**Figure 1:**
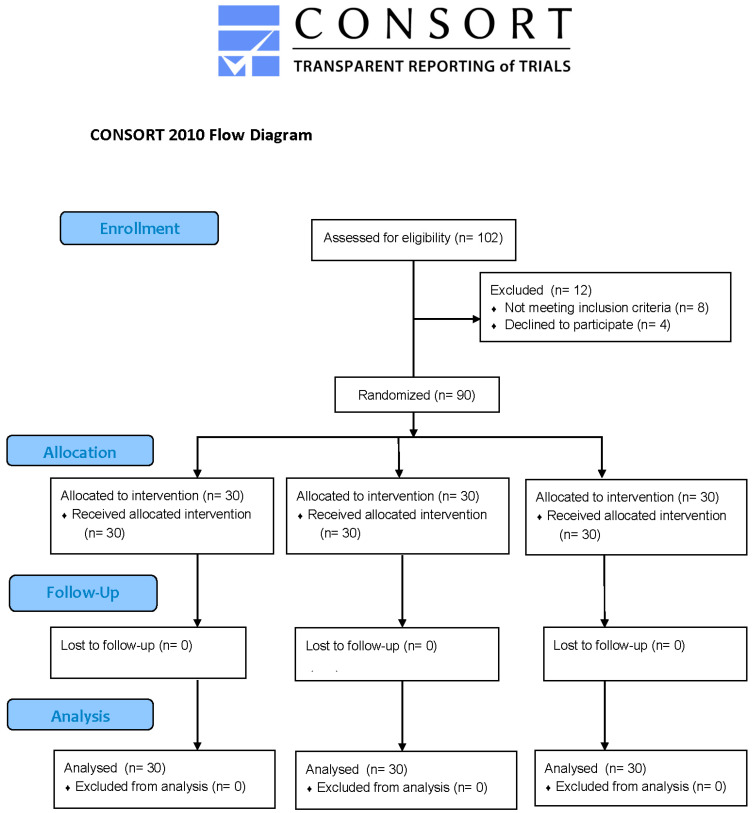
CONSORT flow diagram of patient enrollment, randomization, allocation, follow-up, and analysis. A total of 102 patients were assessed for eligibility, of whom 90 were randomized into three groups (*n* = 30 per group). No patients were lost to follow-up or excluded from the final analysis.

A total of 102 patients were assessed for eligibility, of whom 12 were excluded (8 did not meet the inclusion criteria and 4 declined to participate). Ninety patients were randomized and allocated equally to three groups (*n* = 30 per group). All randomized patients received the allocated intervention, completed the study, and were included in the final analysis, with no losses to follow-up or exclusions after randomization (Figure 1).

Baseline demographic and clinical characteristics were comparable between groups (Table 1). No significant between-group differences were observed with regard to demographic characteristics, baseline pain intensity, comorbidities, ASA physical status, or surgical duration, indicating successful randomization and balanced baseline clinical status across study groups.

### 3.2. Primary Outcome

The time-to-first rescue opioid analgesia was significantly prolonged in both intervention groups compared with the control group (log-rank *p* < 0.0001). The median time-to-first opioid requirement was 7.85 h (95% CI, 7.40–8.50) in the control group, 13.70 h (95% CI, 12.80–14.50) in the dexamethasone group, and 12.40 h (95% CI, 11.20–14.30) in the dexmedetomidine group (Table 2).

**Figure 2:**
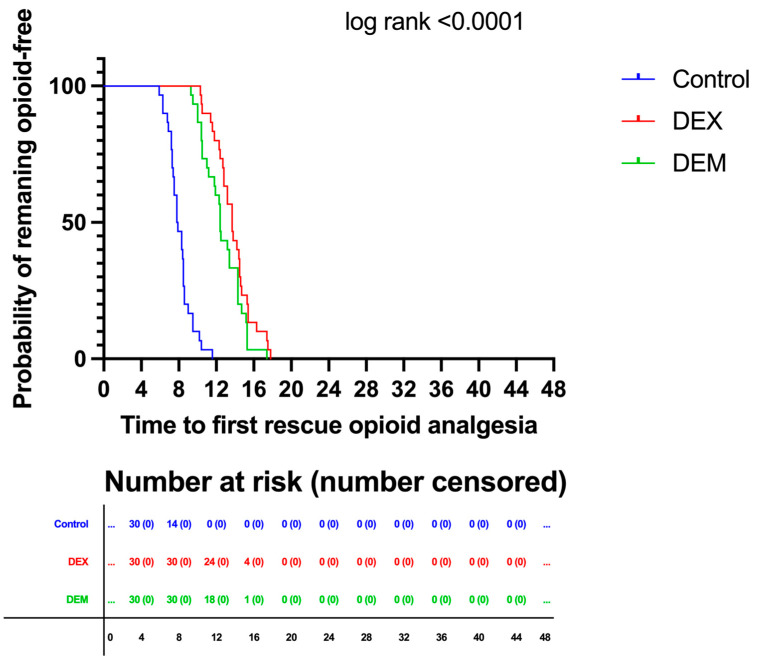
Kaplan–Meier curves for time-to-first rescue opioid analgesia within 48 h postoperatively. All patients required rescue opioid analgesia during the observation period, and all events occurred within the first 24 postoperative hours. Both dexamethasone and dexmedetomidine significantly prolonged the time-to-first opioid requirement compared with control (log-rank *p* < 0.0001).

Kaplan–Meier survival analysis demonstrated a significantly higher probability of remaining opioid-free over time in both the dexamethasone and dexmedetomidine groups compared with the control group (log-rank test, *p* < 0.0001) (Figure 2).

The separation of Kaplan–Meier curves occurred early in the postoperative period and persisted throughout the observation interval, indicating a sustained opioid-sparing effect associated with both adjuvants, particularly dexamethasone.

Cox proportional hazards analysis demonstrated significantly lower hazards of rescue opioid requirement in both the dexamethasone group (HR 0.036, 95% CI 0.017–0.079; *p* < 0.0001) and dexmedetomidine group (HR 0.058, 95% CI 0.028–0.121; *p* < 0.0001) compared with the control group.

### 3.3. Secondary Outcomes

Total opioid consumption within the first 48 h differed significantly between groups (Kruskal–Wallis test, *p* < 0.0001). Post hoc analysis with Dunn’s multiple comparisons test showed that opioid consumption was significantly lower in the dexamethasone group compared with both the control group (*p* < 0.0001) and the dexmedetomidine group (*p* < 0.0001). No significant difference was observed between the control and dexmedetomidine groups (*p* = 0.258).

Two-way repeated-measures ANOVA demonstrated a significant effect of time on postoperative pain intensity (*p* < 0.0001), a significant group effect (*p* < 0.0001), and a significant group-by-time interaction (*p* = 0.0010), indicating different temporal pain trajectories between study groups. Postoperative pain intensity decreased over time in all groups. In the early postoperative period, patients in the dexamethasone group reported consistently lower pain scores compared with both the control and dexmedetomidine groups.

At 4 and 8 h, pain scores were significantly lower in the dexamethasone group compared with the dexmedetomidine group (*p* = 0.014 and *p* < 0.0001, respectively), while no significant difference was observed between the dexamethasone and control groups.

At 12 h, the dexamethasone group had significantly lower pain scores than the control group (*p* < 0.0001), and this difference persisted at 16 h (*p* = 0.0298). Additionally, pain scores remained significantly lower in the dexamethasone group compared with the dexmedetomidine group between 12 and 20 h (all *p* < 0.01).

At 24 h, both the dexamethasone and dexmedetomidine groups showed lower pain scores than the control group (*p* = 0.0045 and *p* = 0.0186, respectively), with no significant difference between the two intervention groups. No significant differences between groups were observed at 36 and 48 h.

The incidence of adverse events was low, and no statistically significant differences were observed between groups. Hypotension occurred in 2 (6.7%) patients in the control group, 2 (6.7%) in the dexamethasone group, and 5 (16.7%) in the dexmedetomidine group (*p* = 0.329). Bradycardia was observed in 0, 1 (3.3%), and 4 (13.3%) patients, respectively (*p* = 0.064). The incidence of PONV was 5 (16.7%), 2 (6.7%), and 4 (13.3%), respectively (*p* = 0.484). No excessive sedation or serious adverse events were observed in any group (Table 3).

## 4. Discussion

The present randomized, double-blind, controlled trial demonstrates that both dexamethasone and dexmedetomidine, when used as perineural adjuvants to erector spinae plane block (ESPB), significantly prolong postoperative analgesia following total knee arthroplasty. However, dexamethasone was associated with a more pronounced and consistent analgesic effect compared with dexmedetomidine. To our knowledge, this is among the few randomized controlled trials directly comparing perineural dexamethasone and dexmedetomidine as adjuvants to lumbar erector spinae plane block in patients undergoing total knee arthroplasty. The present findings, therefore, provide clinically relevant evidence for optimizing fascial-plane block analgesia in contemporary orthopedic anesthesia practice.

The primary finding of this study is the substantial prolongation of time-to-first opioid requirement in both intervention groups compared with the control group. This effect was particularly evident in the dexamethasone group, which demonstrated the longest duration of analgesia [13]. The time-to-event analysis further confirmed these findings, showing a marked reduction in the hazard of requiring rescue opioid analgesia in both adjuvant groups, with a greater effect observed for dexamethasone [14].

In addition to prolonging analgesia, dexamethasone significantly reduced total opioid consumption within the first 48 h postoperatively [15]. In contrast, dexmedetomidine did not significantly reduce opioid consumption compared with the control group [16]. This finding suggests that, despite its effect on delaying the need for rescue analgesia, dexmedetomidine may provide less consistent analgesic benefit over time [17]. This may indicate that dexmedetomidine predominantly delays early postoperative analgesic demand without providing a sustained opioid-sparing effect throughout the entire 48 h observation period.

The analysis of postoperative pain intensity revealed a clear temporal pattern. Dexamethasone provided superior analgesia in the early postoperative period, particularly within the first 20 h, as evidenced by consistently lower NRS scores compared with both the control and dexmedetomidine groups [18]. In contrast, dexmedetomidine did not demonstrate a clinically meaningful advantage over the control group at most time points. Importantly, differences between groups diminished after 24 h, suggesting that the primary benefit of adjuvant use is confined to the early postoperative phase [19].

The safety analysis demonstrated that both adjuvants were well tolerated, with a low incidence of adverse events across all groups. Although not statistically significant, a higher incidence of bradycardia and hypotension was observed in the dexmedetomidine group, which is consistent with the known pharmacological profile of α2-adrenergic agonists [20]. In contrast, dexamethasone was not associated with a statistically significant increase in adverse events compared with the control group [15].

The findings of this study are consistent with previous reports demonstrating that dexamethasone prolongs the duration of peripheral nerve blocks and reduces postoperative analgesic requirements [21]. Additionally, dexamethasone may reduce perineural inflammation and suppress ectopic neuronal discharge, thereby contributing to prolonged sensory blockade and attenuation of central sensitization in the early postoperative period [22]. The mechanism is likely multifactorial, including anti-inflammatory effects, inhibition of ectopic neuronal discharge, and vasoconstriction, which together lead to prolonged local anesthetic action [23]. Dexmedetomidine, on the other hand, exerts its effect primarily through α2-receptor-mediated modulation of nociceptive transmission; however, its clinical efficacy as a perineural adjuvant appears to be less consistent and may be associated with systemic side effects [9].

The clinical relevance of these findings extends beyond analgesia alone. Improved postoperative pain control and reduced opioid requirements may facilitate earlier mobilization, enhance participation in physiotherapy, and support ERAS protocols in total knee arthroplasty [24]. Given the growing emphasis on opioid-sparing perioperative strategies, identification of effective regional anesthesia adjuvants remains highly relevant in modern orthopedic anesthesia [25,26,27].

Several limitations of this study should be acknowledged. First, this was a single-center study, which may limit the generalizability of the findings to other institutions and perioperative protocols. Additionally, although patients up to 100 years of age were eligible for inclusion, the study was not powered to perform subgroup analyses according to advanced age. Age-related differences in the pharmacokinetics and pharmacodynamics of dexamethasone and dexmedetomidine therefore could not be specifically evaluated. Second, although the sample size was adequately powered for the primary outcome, the study may have been underpowered to detect differences in relatively infrequent adverse events and safety outcomes. Although the study was conducted under quadruple-blind conditions, dexmedetomidine-associated hemodynamic effects such as bradycardia or hypotension could theoretically have influenced perceived group allocation among clinical staff. Additionally, intraoperative sedation was not protocolized and was administered at the discretion of the attending anesthesiologist, which may have introduced variability in postoperative sedation-related outcomes such as PONV or perceived sedation levels.

Third, the study evaluated only one dose of dexamethasone and dexmedetomidine; therefore, dose–response relationships could not be assessed. Furthermore, all patients received spinal anesthesia with intrathecal ropivacaine, and the duration of residual spinal sensory and motor blockade was not formally assessed. Variability in spinal block regression may therefore have influenced early postoperative pain assessment and time-to-first opioid measurements. Future studies investigating different concentrations and combinations of adjuvants may provide additional insight into optimization of ESPB analgesia.

Fourth, postoperative follow-up was limited to 48 h, and functional recovery outcomes, including early mobilization, range of motion, rehabilitation milestones, and patient-reported quality-of-recovery measures, were not evaluated. Finally, although lumbar ESPB represents a promising fascial-plane technique for knee arthroplasty, the exact mechanism and spread of local anesthetic remain incompletely understood. In addition, formal sensory block assessment using dermatomal mapping, cold sensation, or pinprick testing was not performed following ESPB because the block was administered after spinal anesthesia. Consequently, the precise extent of sensory coverage and block success could not be objectively verified. Therefore, the observed analgesic effects may have been influenced not only by the regional block itself, but also by systemic absorption of adjuvants or residual spinal anesthesia. Despite these limitations, this study has several strengths, including a randomized, double-blind design, complete follow-up, and robust time-to-event analysis for the primary endpoint. The consistency of findings across multiple outcomes further strengthens the validity of the results.

### 4.1. Clinical Implications

From a clinical perspective, the results suggest that dexamethasone is a more effective adjuvant than dexmedetomidine for prolonging analgesia and reducing opioid consumption when used with ESPB in total knee arthroplasty. Given its superior analgesic efficacy and the absence of a statistically significant increase in adverse events, dexamethasone may represent a promising adjuvant for ESPB in total knee arthroplasty.

### 4.2. Future Research Directions

Future multicenter randomized trials with larger patient populations are warranted to confirm the present findings and further evaluate the optimal adjuvant strategy for ESPB in orthopedic surgery. Additional studies should investigate long-term functional outcomes, rehabilitation parameters, patient satisfaction, and the potential role of combined adjuvant regimens. Furthermore, comparative studies between ESPB and other motor-sparing regional anesthesia techniques within ERAS pathways may help define the precise role of ESPB in total knee arthroplasty.

## 5. Conclusions

Dexamethasone and dexmedetomidine both prolong the duration of analgesia when used as adjuvants to erector spinae plane block in total knee arthroplasty. However, dexamethasone provides superior analgesic efficacy, reflected by longer time-to-first opioid requirement, lower opioid consumption, and reduced early postoperative pain scores. In addition, dexamethasone demonstrated superior analgesic efficacy without a statistically significant increase in adverse events.

## Figures and Tables

**Table 1:** Baseline characteristics. Values are mean with SD and median with IQR.

Variable	Control (*n* = 30)	DEX (*n* = 30)	DEM (*n* = 30)	*p*-Value
Age (years)	67.2 ± 8.5	66.5 ± 9.1	68.1 ± 7.9	0.78
Sex (M/F)	12/18	11/19	13/17	0.88
BMI (kg/m^2^)	29.8 ± 4.2	30.5 ± 4.7	29.9 ± 4.1	0.81
ASA I/II/III	6/18/6	5/19/6	7/17/6	0.92
Duration of surgery (min)	95 ± 15	98 ± 17	96 ± 14	0.74
Preoperative NRS	6.2 ± 1.1	6.0 ± 1.3	6.1 ± 1.2	0.89
Affected side (R/L)	16/14	15/15	17/13	0.83
Hypertension (%)	20 (67%)	18 (60%)	19 (63%)	0.81
Diabetes mellitus (%)	8 (27%)	7 (23%)	9 (30%)	0.78

There were no significant differences in age, sex distribution, body mass index, ASA physical status, duration of surgery, preoperative pain intensity, or comorbidities, including hypertension and diabetes mellitus (all *p* > 0.05).

**Table 2:** Primary and secondary outcomes.

	Control (*n* = 30)	DEX (*n* = 30)	DEM (*n* = 30)	*p*
**Time-to-first rescue opioid analgesia** (hours), median (95% Cl)	7.85 (7.40 to 8.50)	13.70 (12.80 to 14.50)	12.40 (11.20 to 14.30)	**<0.0001**
**Total Opioid Consumption in 48 h** (morphine mEQ), Median (IQR)	47.5 (40.0–53.0)	19.5 (17.0–21.25)	35.0 (30.0–51.25)	**<0.0001** Control vs. DEX 0.2583 Control vs. DEM **<0.0001** DEX vs. DEM
**NRS**				
4 h	2.17 ± 0.53	1.90 ± 0.61	2.33 ± 0.55	0.1752 Control vs. DEX
				0.4591 Control vs. DEM
				**0.0142** DEX vs. DEM
8 h	3.17 ± 0.59	2.83 ± 0.75	3.60 ± 0.50	0.1438 Control vs. DEX
				**0.0092** Control vs. DEM
				**<0.0001** DEX vs. DEM
12 h	3.60 ± 0.72	2.77 ± 0.63	3.50 ± 0.86	**<0.0001** Control vs. DEX
				0.8778 Control vs. DEM
				**0.0012** DEX vs. DEM
16 h	3.30 ± 0.70	2.87 ± 0.57	3.6 ± 0.77	**0.0298** Control vs. DEX
				0.2639 Control vs. DEM
				**0.0003** DEX vs. DEM
20 h	3.23 ± 0.73	2.93 ± 0.74	2.87 ± 0.63	0.2609 Control vs. DEX
				0.0641 Control vs. DEM
				**0.0006** DEX vs. DEM
24 h	3.33 ± 0.66	2.73 ± 0.74	2.87 ± 0.63	**0.0045** Control vs. DEX
				**0.0186** Control vs. DEM
				**0.7335** DEX vs. DEM
36 h	2.67 ± 0.48	2.53 ± 0.51	2.70 ± 0.47	0.5511 Control vs. DEX
				0.9598 Control vs. DEM
				0.3873 DEX vs. DEM
48 h	2.60 ± 0.50	2.43 ± 0.50	2.67 ± 0.48	0.4076 Control vs. DEX
				0.8579 Control vs. DEM
				0.1666 DEX vs. DEM

Data are presented as mean ± SD, median (IQR), or median (95% CI), as appropriate. Comparisons between groups were performed using the Kruskal–Wallis test followed by Dunn’s post hoc test. Post hoc comparisons for opioid consumption: Control vs. DEX (*p* < 0.0001), Control vs. DEM (*p* = 0.258), DEX vs. DEM (*p* < 0.0001). Postoperative pain intensity was additionally analyzed using two-way repeated-measures ANOVA with Geisser–Greenhouse correction. Significant overall effects were observed for group (*p* < 0.0001), time (*p* < 0.0001), and group × time interaction (*p* = 0.0010).

**Table 3:** Adverse events and safety outcomes.

Adverse Event	Control (*n* = 30)	DEX (*n* = 30)	DEM (*n* = 30)	*p*
Hypotension, *n* (%)	2 (6.7)	2 (6.7)	5 (16.7)	0.4941
Bradycardia, *n* (%)	0 (0)	1 (3.3)	4 (13.3)	0.1220
PONV, *n* (%)	5 (16.7)	2 (6.7)	4 (13.3)	0.6107
Excessive sedation, *n* (%)	0 (0)	0 (0)	0 (0)	NA
Neurological symptoms, *n* (%)	0 (0)	0 (0)	0 (0)	NA
Serious adverse events, *n* (%)	0 (0)	0 (0)	0 (0)	NA

Data are presented as numbers (%). Categorical variables were compared using the chi-square test or Fisher’s exact test, as appropriate. *p* values were not calculated for outcomes with zero events in all groups.

## Data Availability

The data presented in this study are available on reasonable request from the corresponding author.
